# Oral health of visually impaired schoolchildren in Khartoum State, Sudan

**DOI:** 10.1186/1472-6831-13-33

**Published:** 2013-07-17

**Authors:** Azza Tagelsir, Ahmed Eltigani Khogli, Nazik Mostafa Nurelhuda

**Affiliations:** 1 Department of Orthodontics, Paediatric Dentistry, & Preventive Dentistry. Faculty of Dentistry, University of Khartoum, Khartoum, The Sudan; 2 Department of Paediatric Dentistry, Faculty of Oral and Dental Medicine, International University of Africa, Khartoum, The Sudan

**Keywords:** Visually impaired children, Oral health, Oral health-related quality of life

## Abstract

**Background:**

Although oral health care is a vital component of overall health, it remains one of the greatest unattended needs among the disabled. The aim of this study was to assess the oral health status and oral health-related quality of life (Child-OIDP in 11-13-year-old) of the visually challenged school attendants in Khartoum State, the Sudan.

**Methods:**

A school-based survey was conducted in Al-Nour institute [boys (66.3%), boarders (35.9%), and children with partial visual impairment (PVI) (44.6%)]. Two calibrated dentists examined the participants (n=79) using DMFT/dmft, Simplified Oral Hygiene Index (OHI-S), dental care index, and traumatic dental injuries (TDI) index. Oral health related quality of life (C-OIDP) was administered to 82 schoolchildren.

**Results:**

Caries experience was 46.8%. Mean DMFT (age≥12, n=33) was 0.4 ± 0.7 (SiC 1.6), mean dmft (age<12, n=46) was 1.9 ±2.8 (SiC 3.4), mean OHIS 1.3 ± 0.9. Care Index was zero. One fifth of the children suffered TDI (19%). Almost one third (29%) of the 11–13 year old children reported an oral impact on their daily performances. A quarter of the schoolchildren (25.3%) required an urgent treatment need. Analysis showed that children with partial visual impairment (PVI) were 6.3 times (adjusted) more likely to be diagnosed with caries compared to children with complete visual impairment (CVI), and children with caries experience were 1.3 times (unadjusted) more likely to report an oral health related impact on quality of life.

**Conclusions:**

Visually impaired schoolchildren are burdened with oral health problems, especially caries. Furthermore, the 11-13 year olds' burden with caries showed a significant impact on their quality of life.

## Background

The prevalence of blind children globally is estimated to be 1.4 million, three-quarters of whom live in the poorest regions of Africa and Asia [1]. In low-income countries, the prevalence of childhood blindness may be as high as 1.5 per 1000 children [2]. Such a high prevalence, alongside poor management of resources may result in huge impacts. Childhood blindness impacts negatively on longevity, with up to 60% of blind children dying within one year of losing their eye sight [3]. Early-onset blindness may impact psychomotor, social, and emotional development thus adversely affecting the visually impaired young child [1].

Childhood blindness in developing countries is a result of acquired factors such as measles, ophathalmia neonatroum, traditional eye medicine, and especially corneal scarring related to malnutrition and vitamin A deficiency [1]. A study conducted in five camps for internally displaced people in Khartoum, Sudan, reported a prevalence of 1.4 per 1000 children suffering from blindness. In this case, the reported leading cause was corneal opacities (40%), from vitamin A deficiency, trauma, or measles. Opacities were followed by amblyopia (32.5%) [4].

Oral health and dental care of the disabled has generally been poorer than the general population [5]. High DMFT/dmft scores were manifest in groups of visually impaired schoolchildren in India (6–12 years, mean DMFT of 4.87) and Riyadh, Saudi Arabia (6-7-years, mean dmft of 6.58, SD 2.02 and 11–12 years, mean DMFT score of 3.89,SD 2.67) [6,7]. Poor oral hygiene, gingivitis and periodontal diseases have been reported among visually impaired children in studies from India [6-8], Iran [9], and Turkey [10]. Mann et al. suggested that this can be due to their inability to visualize the plaque on tooth surfaces resulting in inadequate plaque removal and therefore the progression of dental caries and inflammatory disease of the periodontium [11]. Shetty et al. [8] proposed other factors such as lack of manual-visual coordination and parental supervision, and the child’s reduced concern for his/her appearance [8]. There are very few studies addressing the impact of the severity of visual impairment on oral health of blind children. While all studies were in agreement that children with partial visual impairment have better oral hygiene than those with complete visual impairment, caries experience was not significantly different among the two groups of blind children [10].

However, visually challenged children show better oral health scores when compared to children with other sensory, physical or intellectual disabilities [12-14].

Prevalence of traumatic dental injuries varied among children with visual impairment: 9% in Saudi Arabia, 23.1% in Sao Paulo, Brazil, 24.6% in Kuwait to 32.5% in India [15-18]. Yet, these prevalence figures are less than those reported in young children with physical and mental disabilities [19].

All in all, the impact of visual impairment on oral health is not conclusive in the literature. This study aimed to assess the clinical oral health status in terms of: dmft/DMFT index, Oral Hygiene Index, Traumatic Dental Injuries and dental treatment needs, and secondly to assess the oral health-related quality of life using the Child-OIDP questionnaire in 11-13-year-old attendees of Al-Nour Institute for the visually challenged children in Khartoum State, the Sudan. Moreover, the study aimed to examine the relationships between these clinical variables, sociodemographics and visual impairment.

## Methods

A school-based survey was conducted at Al-Nour Institute for the visually challenged in Khartoum- Bahri (Khartoum north), the only school teaching Braille in Khartoum, Sudan. The Sudanese school system is composed of two levels – primary and secondary. The primary schools include classes from grade 1 to grade 8. Al-Nour school follows the primary school model. Being the only school providing Braille, the age of children attending happens to be very wide (6 – 18 years). As of 2010 this mixed public school had 92 pupils - 61 (66.3%) boys and 31(33.7%) girls. Of the whole school population, 33 pupils were boarders (35.9%).

Field work was conducted between November and December 2010. Data were collected through clinical examination, face to face interviews (personal data and The Child Oral Impacts on Daily Performances (Child-OIDP) questionnaire) and school records.

According to the International Classification of Diseases (Update and Revision 2006) [20], there are four levels of visual function, namely normal vision, moderate visual impairment, severe visual impairment and blindness. Moderate visual impairment combined with severe visual impairment is grouped under the term “*low vision*”: low vision taken together with blindness represents all visual impairments. In this study we have categorized visual impairment into level 2 and level 3 to describe partial visual impairment and level 4 (blindness) to represent complete visual impairment.

Obtained from the school records were the age and visual status, thus categorizing the children into ‘complete’ and ‘partial’ visual impairment; CVI and PVI respectively.

### Clinical examination

Two calibrated paediatric dentists (AT, AE) performed the clinical examination under adequate natural light using a plane mirror and a blunt explorer. Caries was measured using the DMFT/dmft index according to WHO criteria [21]. Dental caries experience was DMFT>0 or dmft>0. It was detected at the cavitaion level only (detectable softened floor, undermined enamel or softened wall). Criteria of “catching” or “retention” of the explorer was not used to detect caries. An explorer was used to remove large debris and to aid in assessing the oral hygiene.

Oral hygiene was assessed using the Simplified Oral Hygiene Index (OHI-S) of Green and Vermillon (1964) [22] - including both components; the Debris index and Calculus index [22]. Score was recorded from six index teeth (all first molars (4), upper right and lower left central incisors (2)) per child. Labial surfaces were examined for all teeth with the exception of the lower molars, where the lingual surfaces were examined. Codes for the Debris index were as follows: 0= Absence of debris or extrinsic stain, 1= debris covering not more than one third of the tooth surface, 2= debris covering more than 1/3 but not more than 2/3 of the tooth surface regardless of the presence of extrinsic stain, 3= Soft debris covering more than two thirds of the examined tooth surface.

The calculus index was scored as follows: 0= No calculus present, 1= Supragingival calculus covering not more than a third of the exposed tooth surface, 2= Supragingival calculus covering more than one third but not more than two thirds of the exposed tooth surfaces or the presence of individual flecks of subgingival calculus around the cervical portion of the tooth or both, 3= Supragingival calculus covering more than two third of the exposed tooth surface or a continuous heavy band of subgingival calculus around the cervical portion of the tooth or both. Accordingly the oral hygiene of each child was classified as good, fair, or poor. Scores for OHI-S values were as follows: poor (≥ 2), fair (1.0 – 1.9) and good (≤ 0.9). For the bivariate analysis the OHI-S the poor and fair categories were combined to describe ‘poor’ oral hygiene.

Dental trauma was measured using the traumatic dental injuries (TDI) index [23]. Codes of the TDI were as follows: code 0=no TDI, code 1=treated TDI, code 2=enamel fracture only, code 3=enamel/dentine fracture, code 4=pulp injury, code 5=tooth missing due to trauma. A code of 9 was given if for any reason a tooth or tooth space could not be scored, or did not warrant a code of 0 to 5.

Treatment needs were categorized into two groups: Urgent treatment need, defined as pain inside the mouth, possible pulpal involvement, or broken or missing restorations with decay, and non-urgent treatment need, defined as any or all of the following: no pain in the mouth; decay present but most likely not involving the pulp; broken restorations with no decay or marginal discoloration, gingivitis or periodontal involvement [24].

### The Child Oral Impacts on Daily Performances (Child-OIDP) Questionnaire

Oral health-related quality of life was measured using the eight- item Child-OIDP questionnaire [25], validated previously in a Sudanese child population [26]. This inventory has the ability to provide information on condition specific impacts whereby the respondent attributes the impacts to specific oral conditions or diseases; thus contributing to the needs assessment and the planning of oral health care services [27]. In the classical questionnaire, tested on Sudanese children in 2008, the participating children were first presented with a list of 16 impairments; toothache, sensitive teeth, tooth decay (hole in teeth), exfoliating primary teeth, tooth space (due to a non-erupted permanent tooth), fractured permanent tooth, colour of tooth, shape or size of tooth, position of tooth, bleeding gum, swollen gum, calculus, oral ulcers, bad breath, deformity of mouth or face, erupting permanent tooth and missing permanent tooth. Criterion and concurrent validity for the 8 item Child-OIDP inventory was demonstrated in that the mean Child-OIDP-sum score increased as children’s self-reported oral health changed from good to bad and from satisfied to dissatisfied. These results were all statistically significant. The questionnaire was re-introduced to 10 of the students to test reproducibility, and the weighted Kohen’s Kappa was 1.0 for all variables

In this study, participants were not prompted on all the 16 impairments. Those that were dropped were: color, shape and size, position, deformity of mouth or face – assuming that the participants could not make a fair judgment based upon their visual challenge.

From the presented impairment list, the schoolchildren selected those that they experienced in the past 3 months. Then, they were asked about the frequency and severity of each of the 8 Child-OIDP items, e.g. ‘*Has your oral health affected your eating habits*, *speaking*, *mouth cleaning*, *relaxing*, *maintaining your emotional state*, *smiling*, *schoolwork and contact with people in the past three months*?’ If the schoolchild responded positively, he/she was asked about the frequency and severity of each impact, e.g. “*How often did this happen*? *How severe was it*?’ A single impact frequency scale for individuals affected on a regular basis was used. The frequency and severity of impacts were scored on a 3 point Likert scale (1–3) as follows: Frequency scores (1) being once or twice a month, (2) three or more times a month, or once or twice a week (3) three or more times a week. Severity scores; 1= little effect, 2= moderate effect and 3= severe effect. Lastly, the children were asked to mention the impairments they thought caused the impact on each performance. A maximum of 3 impairments per impact were recorded.

Children were asked about their perception of and satisfaction with their oral health status. “How do you perceive your oral health?” Possible answers were: 0- I do not have an idea, 1- very good, 2-good, 3-bad, 4-very bad. “Are you satisfied with your oral health?” Possible answers were: 0- I do not have an idea, 1- very satisfied, 2-satisfied, 3-not satisfied, 4-not satisfied at all. Children were asked whether they had visited a dentist in the past.

Information on the children’s parental education and occupation was collected. Children were asked to choose from one of the following categories for education if the parent was alive: No education, primary education, secondary education, tertiary education/university, preschool Quranic education, do not know. These were further combined into two groups: Not educated (no education, primary), and educated (all others). Children who answered ‘don’t know’ were excluded. For occupation the categories were as follows: Not working, housewife (for mothers), teacher, government employee, employee in private sector, business, student, other. This information was combined into two groups, not working (housewife, and not working) and working (all others).

Ethical clearance was obtained from the University of Khartoum ethical committee and consent was obtained from the school authorities and parents. All students who required dental treatment were referred to University based paediatric dental clinics for dental care.

### Statistical methods

These were conducted using SPSS 17.0 (SPSS Inc., 2009). Frequencies, means and crude percentage agreement were computed for descriptive purposes. Cohen’s Kappa (n=10) was applied for test-retest reliability. Binary logistic regression was applied to assess the relationship of oral health variables with socio demographic and visual impairment. The model with caries experience as an outcome is the only one that was statistically significant (p < 0.05).

## Results

### Sample profile

The response rate was 85% (n=79) for the clinical examination, and 89% (n=82) for the Child-OIDP questionnaire. Those children who dropped out were absent from school on the visit day. The clinically examined sample (n=79) consisted of 55 (69.6%) boys and 24 (30.3%) girls. Half of them (n=51, (55.4%)) suffered complete visual impairment. One third lived on campus (n=33, 35.9%). Age range of the study participants was 6–18 years with a mean age of 11.8±SD 3.1. Table 1 describes the socio-demographics of the sampled population

**Table 1:** Frequency distribution (%, n) according to sociodemographic characteristics and oral health problems among Al-Nour Institute attendants

	**Whole population**	**P value**
**Mean age ( years)**	11.8 (±SD 3.1)	-
**Father education**		
“Educated”	82.6	
“No education/primary/deceased”	17.4	.000
**Mother education**		
“Educated”	80.4	
“No education/primary/deceased”	19.6	.000
**Father occupation**		
“Not working/retired/deceased”	19.6	
“ Working”	80.4	.000
**Mother occupation**		
“Not working/housewife/deceased”	58.7	
“Working”	41.3	.095
**Residence**		
Boarding	35.9	
Non-Boarding	64.1	.007
**Perception of oral health**		
“Very good”	70.4	
“Good”	18.5	
“Bad”	4.9	.000
“Very bad”	3.7	
**Satisfaction with oral health**		
“Very satisfied”	76.5	
“ Satisfied”	14.8	
“Not satisfied”	4.9	.000
“Not satisfied at all”	3.7	
**Past dental visit**		
“Never visited”	92.4	.000
“Yes”	7.6	

P-value reports the significance of the Chi-square test comparing the prevalence between groups. Difference considered significant at P < 0.05 (Chi-square test).

Kappa results: during data collection, a group of 10 children (more than 10% of the study sample) was re-examined by both investigators (AE, AT) to assess inter-examiner reliability for dental caries diagnosis. The mean inter-examiner agreement (kappa’s value) was 0.91.

### Caries experience

Caries experience was 46.8%. Mean DMFT (age≥12, n=33) was 0.4 ± 0.7 and significant caries index (SiC) for permanent teeth was 1.6. Mean dmft (age<12, n=46) was 1.9 ± 2.8 and significant caries index for primary teeth was 3.4. Caries experience for deciduous teeth (dmft) was 23.9% and for permanent teeth was 19.6%. The decayed (D,d) component formed the largest contribution to dmft and DMFT.

No significant differences were found when comparing DMFT of boys to girls (mean DMFT was 0.34 ± 0.75 and 0.39 ± 0.92 respectively). Similarly, when comparing boarding and non boarding students, mean DMFT was 0.36 ± 0.82 versus 0.36 ± 0.80, respectively, with no significant difference. Care Index (FT/DMFT and ft/dmft) was zero for all.

A quarter of the schoolchildren (25.3%) required an urgent need for treatment. Deep dental caries with possible involvement of the pulp or related dental abscesses constituted more than 90% of these urgent treatment needs.

In bivariate and multivariate analysis visual impairment was significantly associated with caries experience (Table 2). After adjusting for all variables (Table 2), it was found that children with partial visual impairment (PVI) were 6 times (OR 6.3 95% CI (1.7-22.7)) more likely to be diagnosed with caries over their counterparts with complete visual impairment (CVI).

**Table 2:** Bivariate analysis between the independent variables: socio-demographic and health indicators, and the outcomes: caries experience (DMFT > 0), OHI-S, TDI and C-OIDP with odds ratio (OR) and 95% confidence interval (CI)

	**Caries experience DMFT/dmft>0**		**OHI-S -poor OHI**	**TDI - Trauma present**	**C-OIDP impact >0 (Age 11–13)**
	**OR (95% CI)**		**OR (95% CI)**	**OR (95% CI)**	**OR (95% CI)**
	**Unadjusted**	**Adjusted #**	**Unadjusted**	**Unadjusted**	**Unadjusted**
					
**Age**					
**Under 12**	1	1	1	1	-
12 and above	0.1 (0.0–0.3)	0.07 (0.0–0.3)	0.9 (0.4–2.4)	1.8 (0.6–5.5)	
**Gender**					
**Girls**	1	1	1	1	1
Boys	0.7 (0.3–1.8)	1.1 (0.2–5.4)	0.9 (0.3–2.4)	0.8 (0.2–2.8)	0.8 (0.6–1.1)
**Residence**					
**Non boarders**	1	1	1	1	1
Boarders	0.9 (0.4–2.1)	0.8 (0.2–3.4)	1.0 (0.4–2.6)	0.5 (0.2–1.5)	1.1 (1.0–1.4)
**Mother education**					
**Not educated**	1	1	1	1	1
Educated	0.5 (0.2–1.3)	0.6 (0.2–2.6)	0.6 (0.2–2.0)	2.2 (0.4–10.7)	0.4 (0.0–6.7)
**Father education**					
**Not educated**	1	1	1	1	1
Educated	0.8 (0.3–2.5)	1.8 (0.3–9.1)	0.8 (0.2–2.6)	0.4 (0.1–1.5)	0.3 (0.0–5.6)
**Mother occupation**					
**Not working**	1	1	1	1	1
Working	0.5 (0.2–1.1)	0.7 (0.2–2.4)	0.8 (0.3–2.0)	1.1 (0.3–3.6)	0.8 (0.7–1.1)
**Father occupation**					
**Not working**	1	1	1	1	1
Working	0.8 (0.3–2.1)	0.8 (0.2–3.3)	2.2 (0.8–6.5)	0.5(0.2–1.8)	0.2 (0.0–3.5)
**Oral health**					
Caries experience					
No	-	-	1	1	1
Yes			2.0 (0.8–5.2)	0.3 (0.1–1.2)	1.3 (0.9–1.7)
OHI-S					
Good	1	1	-	1	1
Poor	2.0 (0.8–5.2)	1.9 (0.6–6.7)		0.6 (0.2–1.7)	1.1 (1.0–1.3)
TDI					
No trauma	1	1	1	-	1
Present	0.3 (0.1–1.2)	0.8 (0.2–4.0)	0.6 (0.8–1.7)		1.0 (0.8–1.0)
**Visual impairment**					
Complete (CVI)	1	1	1	4.3 (1.1–16.5)*	1.3 (0.4–5.0)
Partial (PVI)	3.3 (1.4–8.1)*	6.3 (1.7–22.7)*	3.0 (1.1–8.2)*	1	1

Binary logistic regression model for caries, adjusting for all variables illustrated above.

*p < 0.01.

# Logistic regression model: p = 0.001, chi square = 30.4, Nagelkerke R^2^ = 0.43.

### Oral hygiene

Mean OHI-S was 1.3 ± 0.9 for the whole sample. OHI-S values were grouped into poor (≥ 2), fair (1.0 – 1.9) and good (≤ 0.9). Of the whole sample only 21.5% had poor oral hygiene (OH), 43% had fair OH and 35.4% had good OH. The prevalence frequency percentages of OHI-S categories (good, fair, and poor) were compared according to gender, visual impairment, caries experience, residence and dental trauma. This revealed that majority of boarders (41.9%) had significantly poorer oral hygiene (OHI-S) when compared to non-boarders (p =0.001) and more boys had poor oral hygiene than girls (p=0.03). No significant determinants of OHI-S were found in this sample (Table 2).

### Traumatic dental injuries (TDI)

In total, one fifth of the children suffered a traumatic dental injury (19%). Frequency percentages of TDI were as follows: 72% had mild TDI (code2) (n=18 teeth), and 28% had severe TDI (code3 to code5) distributed as follows: 16% had enamel- dentin fracture (n=4 teeth), 8% had TDI involving the pulp (n=2 teeth), and only one tooth was missing due to TDI (4%). The maxillary permanent central incisors were the most common teeth involved (80%) (n=20 teeth).

Regarding the relationship between dental trauma and visual impairment, children with CVI were 4 times more likely to experience dental trauma (p=0.027) than children with PVI (OR 4.3 95% CI (1.1-16.5) (Table 2).

### The Child Oral Impacts on Daily Performances (Child-OIDP)

Only 15.9% of the whole examined population (n=13) reported an oral impact on their daily performance. The most reported impairment associated with oral health related impact on daily performance was toothache followed by sensitivity. As for the age group 11–13, 8 out of 28 (29%) reported oral health related impacts on quality of life. The most common reported impairments were toothache followed by exfoliating teeth. Bivariate analysis did not show any significant associations between the impact on quality of life and the clinical and non clinical parameters.

Bivariate analysis was run for all examined outcomes – Caries, OHI-S, TDI and C-OIDP impact. However, the logistic regression model was used only for caries. The examined variables could not be fit into a statistically significant regression model with the other outcomes, implying that the measured variables did not explain the outcome.

## Discussion

Al-Nour Institute, being the only comprehensive school for the visually impaired in Khartoum, the capital of the Sudan, is formed of a diverse group of students in terms of ethnicity and socio-economic status. Although the authors are aware that the sample population of this report may not be representative of all blind children in Sudan, it should be emphasized that the subjects of the study belonged to the only teaching institution for the blind children in the capital Khartoum. In this population, the proportion of children with caries experience was found to be higher (twice as much) than the reported proportion among non- disabled 12-year-old Sudanese school children (24%) [28]. On a global level, the proportion of caries-free children (53.2%) in this study was higher than those reported from comparable population in Turkey (26.4%) [10], India (1.5%) [8] and Kuwait (35.5%) [13]. Differences in the proportion of caries-free children could be attributed to differences in dietary patterns and accessibility to sweet snacks of these populations. Shetty and co-authors, in the latter study, stated that the higher consumption of sweets and in between snacking, in addition to the daily serving of a sweet dish at school could be the reason of the very high proportion of blind children with decayed teeth [8]. On the other hand, the caries severity (DMFT) of the study participants was found to be similar to the reports from the most recent study from Sudanese schoolchildren (DMFT 0.4 SD 0.92) [28]. Other studies on visually impaired children reported higher caries severity (DMFT) [6,12,13]. Caries severity reported in this study might have been diluted in the wide age range of this sample. Variations in examination procedures may also be a contributing factor. For instance, Reddy and Sharma [6] used sharp probe for examination while no probe was used for caries detection in the present study. Shyama et al. [13] examined subjects facing a window, not under direct sunlight as in the current study. While Significant caries index (SiC) for children above 12 years old in this study was below 3, thus in-keeping with global oral health goal for 12-year-olds for the year 2015 [29], SiC for children below 12 years old was above 3 (3.4). However, there is no WHO target for the SiC levels for primary teeth of children less than 12 years old. Since there is no such a target, no comparison of the study finding with a standard value was possible. An interesting finding in this study was that children with PVI were more likely to be diagnosed with caries as opposed to their counterparts (CVI). Although there is scarce evidence on the relationship between the degree of blindness and caries experience, Desai et al., in 2001 reported a significant inverse association between the level of independence for self-care activities in children with disabilities and number of decayed teeth and DMFT/dmft index [30]. This finding, which is challenged by the findings of the current study, supports the assumption that children with CVI-being less independent than children with PVI- have more carious teeth. Moreover, a recent study in 2012 by Bekiroglu et al. revealed no significant association between the degree of blindness of 7–16 years old visually impaired students and their caries experience [10].

In this study, most of the children were found to have a fair standard of oral hygiene. However, considerable percentage of boarders in this study had significantly poorer oral hygiene when compared to non-boarders. In contrast to our findings, most studies of visually impaired children report fair to poor levels of oral hygiene [8,9,12]. In addition to the common factors such as lack of manual-visual coordination of the blind child and the child’s reduced concern for his/her appearance [6], The suboptimal levels of oral hygiene of those living in campus in this study population could be attributed to lack of assistance or supervision of care givers during performance of oral hygiene practices.

Traumatic dental injuries are common among schoolchildren with a prevalence ranged from 6.19% to 58.6% [31]. Baghdady et al. [32], found a 5.1% prevalence of TDI among non-disabled schoolchildren in Khartoum province. However, no published data are available regarding TDI among attendees of Sudanese special needs schools. Children with disabilities are considered to be at a higher risk for TDI where prevalence rate as high as 32.5% have been previously reported among visually impaired schoolchildren [18]. In the present study, the significantly higher occurrence of traumatic dental injury among completely blind children (CVI) was in accordance with Bhat et al. [18] and O’Donnell [33]. Relatively high prevalence of TDI among this group (19%) could be attributed to the lack of social inclusion policies both inside and outside the school environment. Although the school floor was levelled all around, several pillars supporting the buildings lined the playground. These clearly formed a risk for accidents. Even though more than 70% of TDI were categorized as mild dental trauma, it is worth mentioning that none of the traumatic dental injuries was treated or even seen by an oral health professional.

Dental care is the most frequently unmet health care need for children with special health care needs [34]. In this study population, almost quarter of the children needed urgent dental treatment. These findings reflect serious lack of access to dental treatment. Barriers to receiving dental care such as cost of the service, transportation, lack of trained and experienced dentists are commonly cited in the literature [35]. Other barriers to equal access to dental treatment for individuals with disabilities include inadequate facilities due to restricted financial resources and complex treatment needs requiring special care or general anaesthesia [36]. All reasons similarly apply to the Sudanese context.

The findings of the present study demonstrated an extensive unmet dental treatment needs (dental caries and dental trauma). Dental care was not a priority in the school. The investigators in this study met with the school teachers and educated them on the importance of oral health care and provided practical guidelines to daily oral hygiene practices and dental treatment options. This report is expected to draw the attention of the authorities to this deprived group, to establish oral preventive and curative programs.

In the literature, a number of oral health- related quality of life (OHRQoL) measures have been developed to assess and describe the oral impacts on people’s quality of life. Five of these instruments were designed to assess the OHRQoL in children specifically. These include the following questionnaires: Child Perception Questionnaire (CPQ _11–14_), the Michigan OHRQoL scale, the Child Oral Health Impact Profile (Child-OHIP), the Early Childhood Oral Health Impact Scale (ECOHIS) and the Child Oral Impact on Daily Performance (Child-OIDP). None of the tools have considered children with disability. For this reason, and for the fact that Child-OIDP was the only tool validated in Arabic and in the Sudan, it was used in this study. Findings from this study emphasize the necessity to construct a questionnaire for oral health related quality of life in children with special needs.

The Child-OIDP questionnaire was adapted at the level of impairment selection. Psychometric properties and further scoring of outcomes were not studied because only 15.9% (n=13) reported an oral impact on their daily performance. The low response could also have been a result of the questionnaire not being designed for challenged children.

The strength of this study is in the representativeness of this survey to school children with visual impairment in Sudan. This is the first report examining oral health of the visually impaired in Sudan. A limitation in the study was the use of DMFT to measure caries experience. This index usually underestimated caries because it measures only frank cavitations [37]. Having summed up DMFT and dmft was another study limitation. However, the main interest was to report on caries experience and its examined determinants, and in that respect the summation was appropriate.

## Conclusion

The findings of this study showed that the caries experience of the visually challenged schoolchildren to be high, with those with partial visual impairment more likely to be diagnosed with caries. This population has extensive dental treatment needs and extremely deficient dental care index. As for the age group 11–13, a significant reported oral health related impacts on quality of life was evident.

## Recommendations

Relevant oral health promotion and treatment programs need to be established urgently. More attention has to be directed by the oral health authorities to establish school- based dental care programs comparable to those in elementary schools of the non-disabled children.

## Competing interests

The authors of this article declare that they have no competing interests.

## Authors’ contributions

All authors contributed to the design, data collection, analysis and write-up of the manuscript. All authors have read and approved the final manuscript.

## Pre-publication history

The pre-publication history for this paper can be accessed here:

http://www.biomedcentral.com/1472-6831/13/33/prepub
